# Acaricidal Efficacy of Diatomaceous Earths on Different Life Stages of *Acarus siro* L. and *Tyrophagus putrescentiae* (Schrank)

**DOI:** 10.3390/insects16070693

**Published:** 2025-07-04

**Authors:** Maria C. Boukouvala, Constantin S. Filintas, Nickolas G. Kavallieratos

**Affiliations:** Laboratory of Agricultural Zoology and Entomology, Faculty of Crop Science, Agricultural University of Athens, 75 Iera Odos str., 11855 Athens, Greece; p1172219@aua.gr (C.S.F.); nick_kaval@aua.gr (N.G.K.)

**Keywords:** natural insecticides, InsectoSec, Fossil Shield, stored-product mites

## Abstract

*Acarus siro* L. and *Tyrophagus putrescentiae* (Schrank) (Sarcoptiformes: Acaridae) are cosmopolitan mite species in food storage and processing environments, infesting a wide variety of commodities. In the current study, the diatomaceous earths (DEs) InsectoSec and Fossil Shield were evaluated for wheat protection against adults, larvae, and nymphs of *A. siro* and *T. putrescentiae*. Both DEs were examined at 200 and 500 ppm at 1, 2, and 5 days post-exposure. The efficacy of both formulations against *A. siro* and *T. putrescentiae* life stages depended on dose and exposure. Mortality of *A. siro* reached 100% in larvae, 99.3% in nymphs, and 95.6% in adults by day 5 at 500 ppm of InsectoSec. Similarly, Fossil Shield achieved almost complete larval mortality (99.3%) of *A. siro* at 500 ppm. For *T. putrescentiae*, 100% mortality was observed for larvae at both doses of InsectoSec and for adults or nymphs at 500 ppm by the fifth day. Fossil Shield caused a similar mortality to larvae, reaching 97.0% and 100%, at 200 and 500 ppm, respectively, after 5 days. Our findings indicate that InsectoSec and Fossil Shield can be used as sustainable management tools against *A. siro* and *T. putrescentiae*.

## Simple Summary

The acaricidal efficacy of two diatomaceous earths (DEs), InsectoSec and Fossil Shield, was investigated against various developmental stages of *Acarus siro* L. and *Tyrophagus putrescentiae* (Schrank) (Sarcoptiformes: Acaridae) on wheat at two dose rates. Both DEs demonstrated dose- and exposure-dependent efficacy against *A. siro* and *T. putrescentiae* at all developmental stages. Additionally, the immature stages of both species (i.e., larvae and nymphs) were more susceptible to DEs than adults. *Tyrophagus putrescentiae* was more sensitive to both DEs than *A. siro*. InsectoSec was more toxic compared to Fossil Shield.

## 1. Introduction

*Tyrophagus putrescentiae* (Schrank) and *Acarus siro* L. (Sarcoptiformes: Acaridae) are among the most prevalent mite pests encountered in food storage and processing environments [1,2]. These species are cosmopolitan in distribution, infesting a broad range of commodities, including raw grains, grain by-products, legumes, oilseeds, flours, dairy products, dried or fresh vegetables and fruits, spices, and animal feed [2,3,4]. Their ability to exploit high-humidity conditions and organic-rich substrates make them especially problematic in poorly ventilated or inadequately maintained facilities [5]. Beyond direct feeding damage, infestations by these mites lead to significant quality degradation due to contamination with cast skins or fecal matter and the accumulation of allergens that can pose serious health risks to consumers and workers [6,7]. Moreover, *T. putrescentiae* and *A. siro* are known to favor the growth of several fungi species that are responsible for contamination of food with their mycotoxins [8]. Notably, *T. putrescentiae* has been observed to feed on various mycotoxin-producing fungi, including *Fusarium guttiforme* and *Penicillium citrinum*, as well as on other medically important species like *Trichophyton mentagrophytes*, facilitating their spread to new storage environments [7].

Stored-product mites of the family Acaridae are microscopic, making early detection difficult. Coupled with their rapid population growth under a wide range of environmental conditions, mites pose a significant threat to food preservation in storage facilities [5,9]. Their control exhibits considerable challenges due to their small size, rapid population growth, and resistance to conventional pesticides [5,10]. Given the increasing concern over chemical pesticide resistance and safety in food storages, there is a need for effective and environmentally friendly alternatives [11,12]. Diatomaceous earths (DEs) are fine dusts composed of the fossilized remains of diatoms, a vast group of unicellular eukaryotic algae that were especially prevalent during the Miocene and Eocene [13,14]. Presently, diatoms are commonly distributed in both freshwater and marine environments, except for hypersaline habitats [15]. These organisms are encased in a silica-rich outer shell, known as a frustule, that persists long after the organism has died and forms the basis of DE deposits [16,17]. For several years, inert materials such as DEs have been extensively studied for their potency in postharvest pest control and have been developed into commercially available products [18,19]. DEs are considered a generally safe material for mammals [20,21]. The mechanism of action of DEs is generally attributed to fine, sharp particles adhering to the insect cuticle, causing abrasion of the protective waxy layer, thereby disrupting the cuticle and leading to death through increased water loss [22].

Research efforts from the last decade evaluating the effectiveness of various commercial DE formulations against *T. putrescentiae* have demonstrated remarkably high mortality rates, even within a short exposure period (5 days). Notably, it was shown that even low doses of DE formulations on treated wheat can cause high mortality of *T. putrescentiae*, supporting their strong acaricidal potential [23]. A recent study by Mahmoud et al. [24] concluded that DEs exhibit strong acaricidal properties that increase with exposure and dose, in both laboratory and semi-field conditions, against a spectrum of detrimental mite species for agriculture. Furthermore, laboratory tests have shown that both dust and wettable powder formulations of DEs can procure complete mortality of storage mites within a day of exposure in controlled environmental conditions [25]. The commercial DE InsectoSec is primarily used as an acaricide for the control of *Dermanyssus gallinae* (De Geer, 1778) (Mesostigmata: Dermanyssidae) on poultry farms [26,27]. Its efficacy has also been evaluated against various stored-product insect pests [26,27]. Fossil Shield is one other commercial DE product that has been previously tested for its effectiveness on cereal grains and pulses against storage beetles [26,27,28,29]. However, no data is available on their efficacy against *T. putrescentiae* and *A. siro.* The present study aimed to evaluate the acaricidal efficacy of InsectoSec and Fossil Shield against *T. putrescentiae* and *A. siro* under laboratory conditions. To better assess the potential of these commercially available DEs as grain protectants against notorious stored-product mites, experiments were conducted on wheat at various exposure intervals and life stages (adults, larvae, and nymphs).

## 2. Materials and Methods

### 2.1. Mite Species

Colonies of *T. putrescentiae* and *A. siro* were initially established in 2004 using specimens collected from stored wheat in facilities across Greece. They have been continuously maintained under complete darkness, at 25 °C temperature, and 80% relative humidity (RH) in the Laboratory of Agricultural Zoology and Entomology of the Agricultural University of Athens [30]. Both mite species were reared in plastic containers (200 mm × 120 mm × 60 mm). *Tyrophagus putrescentiae* was maintained on a diet consisting of bran, flour, and brewer’s yeast in a 40:10:1 weight ratio, while *A. siro* was cultured on a blend of oat flakes, wheat germ, and dried yeast extract at a 10:10:1 ratio. Individuals representing all life stages—larvae, nymphs, and adults—were selected for experimentation based on morphological traits. Specifically, adults were distinguished by their larger size and longer body setae, while larvae were identified by their smaller size and three pairs of legs [31].

### 2.2. DE Formulations

Two commercially available DE formulations were used in the experiments: InsectoSec (Protecta, Peristeri, Greece) is a product originating from marine waters containing 10% food-grade bait, 87% amorphous SiO_2_, ~3% Al_2_O_3_, 1% Fe_2_O_3_, and <1% CaO, MgO, TiO_2_, and P_2_O_3_. The particle size of InsectoSec is about 8.2 μm [27]. Fossil Shield 90.0 (Fossil Shield, Bein GmbH, Eiterfeld, Germany) contains 87% amorphous SiO_2_, 3% aerosol, 4.9% Al, 4.7% Fe, 1.3% K, 0.8% Ca, 0.8% Mg, and 0.3% Na. The particle size of Fossil Shield is 9.5 µm [27].

### 2.3. Grain Commodity

Pesticide-free hard wheat (*Triticum durum* Desf., var. Mexa) was used for the tests. The moisture content (m.c.) of the grain was standardized to 16%, as measured with a Mini GAC Plus moisture meter (Dickey-John Europe S.A.S., Colombes, France). Depending on the initial moisture level, the wheat was either dried in an oven at 50 °C or moistened with distilled water to reach the desired level, following the procedure outlined by [32].

### 2.4. Bioassays

Both formulations were evaluated at two concentrations, 200 and 500 ppm, which correspond to 0.2 and 0.5 g DE/kg wheat, respectively. For each treatment, 1 kg batches of wheat were placed into cylindrical glass containers and thoroughly mixed with each DE dose by shaking the jars manually for two minutes to ensure uniform coverage. An untreated 1 kg batch served as a control. From each treatment and control group, three samples (1 g) were collected using individual scoops (1 per jar) and transferred into small, inverted truncated conical plastic vials (measuring 3.5 cm across the top, 2.5 cm across the bottom, 3.9 cm in height, and 4.2 cm slant height). Each portion was weighed using a Precisa XB3200D compact balance (Alpha Analytical Instruments, Gerakas, Greece) on a fresh thin plastic layer for every sample. To provide adequate airflow, each vial was sealed off with a lid that had a central opening (1.5 cm in diameter) covered with gauze. The inside edge of each vial was coated with polytetrafluoroethylene (60% aqueous dispersion; Sigma-Aldrich, Taufkirchen, Germany) to prevent mites from escaping. Fifteen individuals (i.e., larvae, nymphs, or adults) of either *A. siro* or *T. putrescentiae* were separately introduced into each vial. The vials were then placed in incubators maintained at 25 °C and 80% RH. This humidity level corresponds to the wheat moisture content of approximately 16%, as previously described [33,34]. Mite mortality was assessed after 1, 2, and 5 days of exposure using an Olympus SZX9 stereomicroscope (Bacacos S.A., Athens, Greece). The experiment followed a completely randomized block design, including three sub-replicates per treatment and three replicates overall, each involving freshly prepared wheat, new vials, and mite individuals.

### 2.5. Data Analysis

Since mortality in the control group remained below 5%, no adjustment was deemed necessary for the results. Before analysis, the data set was converted to log (x + 1) to obtain the standard variance of the data [35,36]. The data were analyzed using a repeated measures statistical model, with separate analyses performed for each mite species and developmental stage (larvae, nymphs, and adults) as described by Sall et al. [37]. The exposure intervals served as the repeated factor, while mortality rates was the response variable. The main effects considered in the model were the applied dose and the type of DE formulation, along with their interactions. All statistical procedures were carried out using JMP version 11 [38]. Mean comparisons were performed using the Tukey–Kramer HSD test at a significance level of 0.05 [39]. A two-tailed t-test with n-2 df and a significance level of 0.05 [40] was used to compare the two concentrations in each treatment for each life stage or species tested.

## 3. Results

### 3.1. Effectiveness of DEs Against A. siro

For *A. siro* larvae and adults, only the intercept and dose were significant, whereas for nymphs, the intercept, DE formulation, and dose were significant between exposure intervals (Table 1). Within the exposure intervals, exposure and exposure × dose were significant for all life stages of *A. siro* (Table 1). InsectoSec and Fossil Shield showed dose- and exposure-dependent efficacy against *A. siro* at all developmental stages. One day post-exposure, larval mortality was low for both formulations, reaching 32.6% at 500 ppm of InsectoSec (Table 2). On the second day of the experiments, moderate mortality was observed, ranging between 49.6 and 64.4%. At 5 days post-exposure, 100.0% mortality was recorded at 500 ppm of InsectoSec while almost complete mortality (99.3%) was observed in Fossil Shield at the same dose.

Nymphal mortality was slightly lower than larval mortality and slightly higher than adult mortality throughout the tests (Table 2). On the first day of observations, both DEs showed low mortality rates in both doses, ranging from 18.5 to 27.4%. After 2 days, a significant increase in mortality was recorded in all dose/formulation combinations, reaching 58.5%. At 5 days post-exposure, mortality increased significantly, with InsectoSec and Fossil Shield killing 99.3% and 92.6% of exposed nymphs, respectively, at 500 ppm.

Concerning the adult stage, mortality remained lower in comparison to both immature life stages (Table 2). On the first day of observations, both formulations provided low mortality, not exceeding 26.0%. One day later, mortality increased, reaching 53.3 and 54.8% in wheat treated with 500 ppm of Fossil Shield and InsectoSec, respectively. Five days post-exposure, mortality of both DEs and in both doses ranged from 80.7 to 95.6%.

### 3.2. Effectiveness of DEs Against T. putrescentiae

For larvae and adults of *T. putrescentiae*, intercept, DE formulation, and dose were significant, while for nymphs, intercept and dose were significant between exposure intervals (Table 1). Within exposure intervals, for all life stages of *T. putrescentiae*, exposure and exposure × dose were significant (Table 1). The efficacy of both DE formulations against *T. putrescentiae* developmental stages depended on both dose and exposure. One day after exposure, larval mortality remained low for both formulations, ranging from 24.4 to 34.8% (Table 3). One day later, mortality increased to moderate levels, not exceeding 63.7 and 66.7 in Fossil Shield and InsectoSec treatments, respectively. At 5 days post-exposure, complete mortality (100.0%) was recorded for both doses of InsectoSec and 500 ppm of Fossil Shield, while at 200 ppm, mortality was almost complete (97.0%).

Concerning *T. putrescentiae* nymphs, mortality was slightly higher than that of adults but lower than that of larvae during the experiment (Table 3). After 1 day of exposure, mortality was low at both formulations and doses, not exceeding 31.1%. On the second day of observations, mortality ranged at moderate levels between 44.4 and 62.2%. At the last day of the experiments, mortality increased notably in all tested combinations (dose/formulation), killing all exposed nymphs at 500 ppm of InsectoSec. Fossil Shield caused 97.0% mortality at 500 ppm, while at 200 ppm, mortality was 84.4% and 86.7% for Fossil Shield and InsectoSec, respectively.

Regarding *T. putrescentiae* adults, mortality remained lower than that observed for the larval and nymphal stages (Table 3). On the first day of the tests, both DEs resulted in low mortality rates, reaching 23.0 and 28.9%, in wheat treated with Fossil Shield and InsectoSec, respectively. On the second day, an increase in mortality was observed, ranging from 40.7 to 60.0%. After 5 days, complete (100%) mortality was recorded at 500 ppm of InsectoSec and 91.1% at the same dose rate of Fossil Shield, while at 200 ppm, mortality reached 83.7%.

## 4. Discussion

The development of alternative means in stored-product protection has included considerable research efforts about DEs the last decades. Several formulations of DEs are registered in different countries for application, at reduced doses, on durable grain commodities and on surfaces in empty storage facilities (silos and bins) to manage pests at the postharvest stage [41]. This study demonstrated that InsectoSec and Fossil Shield were effective against *A. siro* and *T. putrescentiae*, showing dose- and time-dependent mortality. After 5 days, both formulations caused substantial mortality across the tested life stages, especially at the highest dose. Furthermore, InsectoSec demonstrated higher efficacy against the mobile life stages of both mites than Fossil Shield. Regarding *A. siro*, InsectoSec led to complete larval mortality and >95% nymphal and adult mortality, while Fossil Shield was slightly less effective. Similarly, in *T. putrescentiae*, InsectoSec led to complete larval mortality at both tested doses, outperforming Fossil Shield, remaining effective but weaker than Fossil Shield at the lower dose. Adarkwah et al. [26,27] reported a similar trend in the efficacy of InsectoSec and Fossil Shield against the chrysomelid *Callosobruchus maculatus* (F.) (Coleoptera) and the tenebrionid *Tribolium castaneum* (Herbst) (Coleoptera). For instance, after 21 days of exposure, 87.8% mortality was reported at 500 ppm of Fossil Shield vs. 100% mortality at 500 ppm of InsectoSec against *T. castaneum* [27]. The pesticidal efficacy of DEs is influenced by several parameters, such as the geographical source of DE, the composition, and, most importantly, its physical and chemical properties, including particle size, shape, and silica concentration [41]. Smaller particle sizes (< 45 μm) enhance the efficacy of DE formulations against arthropod pests by promoting higher adherence and increased abrasion of the insect cuticle [42]. In addition, the particle shape and size of DEs influence their ability to absorb cuticular lipids and induce dehydration in arthropods [18]. Both formulations used in this study contain the same percentage of amorphous SiO_2_ (i.e., 87%), indicating that this characteristic did not affect their efficacy. The particle size of the tested DEs may have played a role in their effectiveness, since InsectoSec, which has a smaller mean particle size (8.2 μm), exhibited slightly higher efficacy than Fossil Shield (9.5 μm).

Although DEs are recognized as a valuable and safe tool for managing arthropod pests in storage environments, their application rate must be carefully considered due to potential negative effects on grain quality [43]. One of the main concerns associated with their use is related to the impact on bulk density [43]. The general consensus emerging from the literature is that DE formulations should not be applied at rates exceeding 1000 ppm [18,43]. This limit is considered a compromise between effectively controlling arthropod pests and maintaining grain quality. Notably, in this study, the DE concentrations applied were well below the commonly reported upper limit of 1000 ppm, yet proved to be highly effective, with almost complete mortality observed even at 200 ppm after 5 days of exposure (*T. putrescentiae* larvae exposed to InsectoSec). However, in most of the tested cases (mite species/life stage/DE/dose), exposure greater than 5 days is required. Similarly, Iatrou et al. [23] reported that more than 5 days of exposure is required to achieve 100% mortality of *T. putrescentiae* exposed to wheat treated with either PyriSec or SilicoSec at two doses (0.2 and 0.5 g DE/kg). Palyvos et al. [44] evaluated ≥500 ppm of SilicoSec and documented that adult mortality of *T. putrescentiae* increased depending on exposure and dose. For instance, at 500 ppm, complete mortality of *T. putrescentiae* adults was achieved after 7 days of exposure, while at 1000 and 2000 ppm, all exposed individuals were deceased within 48 hours [44].

Our findings suggest that while *A. siro* and *T. putrescentiae* are susceptible to DE exposure, the two species responded slightly differently. Notably, *T. putrescentiae* exhibited relatively higher overall mortality to InsectoSec and Fossil Shield than *A. siro*. A similar trend has been reported by Kavallieratos et al. [30] when both species were exposed to treated wheat with three zeolites. The variability in mite susceptibility to DEs has been previously documented. For instance, *A. siro* was found to be more susceptible to Diasecticide and SilicoSec than *Lepidoglyphus destructor* (Schrank) (Astigmata: Glycyphagidae) [45]. In contrast, Cook and Armitage [46] found that *L. destructor* was less susceptible to Dryacide than *A. siro*. According to Nesvorná and Hubert [47], *L. destructor* was the most susceptible to the Detia DE formulation, followed by *A. siro* and *T. putrescentiae*. Mahmoud et al. [24] noted a higher DE susceptibility of *Rhizoglyphus echinopus* (Fumouze & Robin) (Astigmata: Acaridae) than *T. putrescentiae*. The high susceptibility to DE was also observed when SilicoSec was evaluated against *T. putrescentiae* and the predatory mite *Cheyletus malaccensis* Oudemans (Prostigmata: Cheyletidae), with the latter being more tolerant to DE [44]. The high survivability of the *C. mallaccensis* was attributed to the different structure of its cuticle compared to the soft-bodied *T. putrescentiae* [44]. Therefore, slight differences documented in our bioassays could be similarly attributed to species-specific cuticle composition and thickness. However, these differences could be a random variation; thus, generalizations should be avoided.

The long-term effects of DEs on population growth have been previously evaluated for three species of stored-product mites. DE treatments up to 250 ppm were shown to significantly suppress population expansion over 21 days, with sensitivity varying between species [47]. These findings suggest that even at low doses, DEs can exert a sustained inhibitory effect on mite reproduction and growth, despite not causing immediate mortality. In contrast, the present study, by evaluating the lethal effects of DE exposure over a shorter exposure period (5 days), did show complete mortality in many cases. This comparison highlights the dual potential for both rapid control and long-term population management, depending on the duration of exposure and dosage. Interestingly, the results of the current research revealed clear differences in susceptibility to InsectoSec and Fossil Shield across life stages of *A. siro* and *T. putrescentiae*, with larvae generally exhibiting higher mortality rates, followed by nymphs and adults. Similarly, the immatures of *T. putrescentiae* and *R. echinopus* were more sensitive to DEs than adults [23,24]. A possible explanation of the differences in susceptibility among the life stages of the species examined here may be the variations in cuticle thickness and composition among developmental stages. Thus, this response observed in larvae suggests that targeting early developmental stages of mites may enhance the success of DE when incorporated in pest management strategies.

The climatic conditions play an important role in the survival of *A. siro* and *T. putrescentiae* [48,49]. Although the experiment of this study was conducted under less suitable abiotic conditions of temperature and RH for long storage periods, they are common in tropical and subtropical regions [50]. Furthermore, elevated temperature and moisture conditions in hot areas within storages favor the development of mites even when the open-air conditions are unfavorable for their growth [51]. Therefore, control measures of mites should include insecticidal treatments in combination with monitoring of temperature/RH in variable points of the facility and in the grain mass, regulation of humidity, improved sanitation, and cooling [52].

## 5. Conclusions

The tested DE formulations are highly promising non-chemical alternative means to protect stored wheat from *A. siro* and *T. putrescentiae* infestations. Their high efficacy, combined with a favorable safety profile for both humans and the environment, supports the integration of the commercially available DEs InsectoSec and Fossil Shield into the sustainable management of noxious mites of stored grains. Although it is well-established that various biotic and abiotic factors influence the efficacy of DEs, there is a need for further scientific investigation about their meticulous application against stored-product mites. For example, both tested DEs were effective in elevated temperature, RH, and moisture content conditions. Whether their performance is altered in reduced abiotic conditions that support the growth of mites remains to be investigated. The optimization of DE application strategies is crucial to maximize their potential as reliable tools in grain protection.

## Figures and Tables

**Table 1:** MANOVA parameters presenting the main effects and their interactions leading to the observed mortalities of *Acarus siro* and *Tyrophagus putrescentiae* larvae, nymphs, and adults, between and within exposure intervals (error df = 32 for all species and developmental stages).

Source		*A. siro*						*T. putrescentiae*					
		Larvae		Nymphs		Adults		Larvae		Nymphs		Adults	
	df	*F*	*p*	*F*	*p*	*F*	*p*	*F*	*p*	*F*	*p*	*F*	*p*
All between													
Intercept	1	39,950.4	<0.01	21,886.7	<0.01	16,451.4	<0.01	70,870.4	<0.01	30,232.1	<0.01	28,633.6	<0.01
DE formulation	1	3.9	0.06	4.4	0.04	1.4	0.25	5.4	0.03	3.4	0.08	6.2	0.02
Dose	1	17.6	0.01	13.9	0.01	17.8	0.01	24.3	<0.01	24.7	<0.01	34.9	<0.01
DE formulation × dose	1	0.1	0.88	0.1	0.87	0.2	0.65	0.1	0.95	0.1	0.95	0.1	0.72
Within interactions													
Exposure	2	483.7	<0.01	602.2	<0.01	579.8	<0.01	808.9	<0.01	1186.2	<0.01	592.8	<0.01
Exposure × DE formulation	2	1.3	0.28	2.0	0.15	0.8	0.47	1.7	0.20	2.8	0.08	1.0	0.39
Exposure × dose	2	7.9	0.01	4.1	0.03	4.3	0.02	12.4	0.01	6.8	0.01	8.0	0.01
Exposure × DE formulation × dose	2	0.1	0.91	2.2	0.12	3.2	0.06	0.8	0.45	1.3	0.29	1.1	0.34

**Table 2:** Mean (% ± SE) mortality of *A. siro* larvae, nymphs, and adults on wheat treated with two DE formulations (InsectoSec and Fossil Shield) at two dose rates (200 and 500 ppm) and three intervals (1, 2, and 5 days). Within each column, no significant differences were observed in the means with the same lowercase letters (df = 5, 53 Tukey HSD test at *p* < 0.05). For each exposure, within each developmental stage, asterisks indicate significant differences (df = 16; two-tailed *t*-test at *p* = 0.05). Where no asterisks exist, no significant differences were recorded.

	Stage	Larvae				Nymphs				Adults			
	Dose	200 ppm	500 ppm			200 ppm	500 ppm			200 ppm	500 ppm		
DE	Exposure			*t*	*p*			*t*	*p*			*t*	*p*
InsectoSec	1 day	25.2 ± 2.2 c	32.6 ± 1.3 c *	2.7	0.02	21.5 ± 1.5 c	27.4 ± 1.7 c *	2.5	0.02	16.3 ± 1.6 c	25.9 ± 2.8 c *	2.8	0.01
	2 days	52.6 ± 2.1 b	64.4 ± 1.1 b *	4.6	0.01	47.4 ± 2.1 b	58.5 ± 2.4 b *	3.4	0.01	42.2 ± 2.2 b	54.8 ± 3.8 b *	2.7	0.01
	5 days	96.3 ± 2.3 a	100.0 ± 0.0 a	1.6	0.13	84.4 ± 2.5 a	99.3 ± 0.7 a *	5.4	<0.01	81.5 ± 2.4 a	95.6 ± 2.2 a *	4.2	0.01
Fossil Shield	1 days	22.2 ± 2.2 c	27.4 ± 2.1 c	1.5	0.14	18.5 ± 2.2 c	22.2 ± 2.2 c	1.2	0.27	15.6 ± 1.6 c	20.7 ± 2.1 c	2.0	0.06
	2 days	49.6 ± 1.6 b	61.5 ± 3.1 b *	3.2	0.01	42.2 ± 2.2 b	56.3 ± 3.7 b *	3.0	0.01	39.3 ± 2.1 b	53.3 ± 3.7 b *	3.3	0.01
	5 days	93.3 ± 1.6 a	99.3 ± 0.7 a *	3.4	0.01	83.7 ± 3.5 a	92.6 ± 2.6 a	2.2	0.06	80.7 ± 3.4 a	89.6 ± 2.3 a *	2.1	0.05
	*F*	98.2	135.6			102.0	97.1			141.6	65.5		
	*p*	<0.01	<0.01			<0.01	<0.01			<0.01	<0.01		

**Table 3:** Mean (% ± SE) mortality of *T. putrescentiae* larvae, nymphs, and adults on wheat treated with two DE formulations (InsectoSec and Fossil Shield) at two dose rates (200 and 500 ppm) and three intervals (1, 2, and 5 days). Within each column, no significant differences were observed in the means with the same lowercase letters (df = 5, 53 Tukey HSD test at *p* < 0.05). For each exposure, within each developmental stage, asterisks indicate significant differences (df = 16; two-tailed *t*-test at *p* = 0.05). Where no asterisks exist, no significant differences were recorded. No statistical analysis was carried out where dashes exist.

	Stage	Larvae				Nymphs				Adults			
	Dose	200 ppm	500 ppm			200 ppm	500 ppm			200 ppm	500 ppm		
DE	Exposure			*t*	*p*			*t*	*p*			*t*	*p*
InsectoSec	1 day	26.7 ± 1.8 c	34.8 ± 1.5 c *	3.7	0.01	23.7 ± 2.5 c	31.1 ± 1.1 c *	2.6	0.02	18.5 ± 1.0 c	28.9 ± 2.0 c *	3.3	0.01
	2 days	55.6 ± 1.1 b	66.7 ± 2.2 b *	4.7	0.01	50.4 ± 3.5 b	62.2 ± 1.1 b *	3.2	0.01	45.2 ± 1.5 b	60.0 ± 2.2 b *	5.5	<0.01
	5 days	100.0 ± 0.0 a	100.0 ± 0.0 a	-	-	86.7 ± 4.3 a	100 ± 0.0 a *	3.0	0.01	83.7 ± 1.6 a	100.0 ± 0.0 a *	9.0	<0.01
Fossil Shield	1 day	24.4 ± 1.9 c	29.6 ± 1.6 c	2.1	0.06	20.7 ± 1.7 b	25.9 ± 0.7 d *	2.6	0.02	16.3 ± 2.0 c	23.0 ± 2.0 c *	2.2	0.04
	2 days	51.1 ± 2.5 b	63.7 ± 2.3 b *	3.6	0.01	44.4 ± 2.2 c	59.3 ± 2.1 b *	4.9	0.01	40.7 ± 1.7 b	55.6 ± 2.7 b *	4.5	0.01
	5 days	97.0 ± 1.2 a	100.0 ± 0.0 a *	2.5	0.02	84.4 ± 1.9 a	97.0 ± 1.2 a *	5.4	<0.01	82.2 ± 1.9 a	91.1 ± 2.2 a *	3.0	0.01
	*F*	159.7	243.0			75.1	480.9			131.9	84.7		
	*p*	<0.01	<0.01			<0.01	<0.01			<0.01	<0.01		

## Data Availability

The original contributions presented in this study are included in the article. Further inquiries can be directed to the corresponding author.

## Abbreviation

The following abbreviation is used in this manuscript:

DE	diatomaceous earth
